# Ketogenic Diets and Chronic Disease: Weighing the Benefits Against the Risks

**DOI:** 10.3389/fnut.2021.702802

**Published:** 2021-07-16

**Authors:** Lee Crosby, Brenda Davis, Shivam Joshi, Meghan Jardine, Jennifer Paul, Maggie Neola, Neal D. Barnard

**Affiliations:** ^1^Physicians Committee for Responsible Medicine, Washington, DC, United States; ^2^Brenda Davis Nutrition Consulting, Kelowna, BC, Canada; ^3^Department of Medicine, New York University Grossman School of Medicine, New York, NY, United States; ^4^Department of Medicine, New York City Health + Hospitals/Bellevue, New York, NY, United States; ^5^College of Liberal and Professional Studies, University of Pennsylvania, Philadelphia, PA, United States; ^6^School of Public Health, Loma Linda University, Loma Linda, CA, United States; ^7^Adjunct Faculty, Department of Medicine, George Washington University School of Medicine and Health Sciences, Washington, DC, United States

**Keywords:** ketogenic diet, very-low-carbohydrate diet, low-carbohydrate diet, obesity, disease prevention

## Abstract

Very-low-carbohydrate ketogenic diets have been long been used to reduce seizure frequency and more recently have been promoted for a variety of health conditions, including obesity, diabetes, and liver disease. Ketogenic diets may provide short-term improvement and aid in symptom management for some chronic diseases. Such diets affect diet quality, typically increasing intake of foods linked to chronic disease risk and decreasing intake of foods found to be protective in epidemiological studies. This review examines the effects of ketogenic diets on common chronic diseases, as well as their impact on diet quality and possible risks associated with their use. Given often-temporary improvements, unfavorable effects on dietary intake, and inadequate data demonstrating long-term safety, for most individuals, the risks of ketogenic diets may outweigh the benefits.

## Introduction

Very-low-carbohydrate (ketogenic) diets have been promoted for weight loss and, less commonly, for other health reasons. This review summarizes the effects of a ketogenic diet on health conditions for which it has been promoted, as well as potential long-term effects on health.

The term “ketogenic diet” generally refers to a diet that is very low in carbohydrate, modest in protein, and high in fat. This mix of fuels aims to induce *ketosis*, or the production of ketone bodies that serve as an alternate energy source for neurons and other cell types that cannot directly metabolize fatty acids. Urinary ketone levels are often used as an indicator of dietary adherence (1).

Various ketogenic diets have been studied, as shown in Table 1. The best defined and studied is sometimes called a “classic” ketogenic diet, referring to a very-low-carbohydrate diet that is generally medically supervised, with a 4:1 or 3:1 ratio, by weight, of dietary fat to combined dietary protein and carbohydrate (2).

**Table 1:** Macronutrient composition of ketogenic diets.

**Diet**	**% Energy from fat**	**% Energy from carbohydrate**	**% Energy from protein**	**References**
“Classic” ketogenic (4:1)	90	2–4	6–8	(2)
“Classic” ketogenic (3:1)	85–90	2–5	8–12	(2)
Modified Atkins diet	60–65	5–10	25–35	(2)
Ketogenic, general (<50 g carbohydrate)	70–80	<10	~10	(3, 4)
Low-carbohydrate (<130 g carbohydrate)	Varies	10–25	Varies	(3, 4)

Other variants allow more protein or carbohydrate (2). Ketogenic diets as typically implemented in scientific studies limit dietary carbohydrate to <50 g per day with varying amounts of fat and protein (3, 4). “Low-carbohydrate diets” refer to carbohydrate intake below the recommended dietary allowance of 130 g/day (3), which may not be low enough to induce ketosis (5).

## Effects on Nutrient Metabolism

During prolonged fasting, some tissues, such as muscle, can directly metabolize free fatty acids released from adipose stores. However, much of this fatty acid is converted into ketones in the liver, which can fuel otherwise-obligate glucose consumers like neurons, minimizing mobilization of body protein for gluconeogenesis. However, to induce the liver to make ketones in the fed state, carbohydrate intake must be minimized and fat intake increased. Protein utilization is also altered on a ketogenic diet; the body shunts as much protein as possible to gluconeogenesis, while the minimum necessary amount is used for tissue repair.

## Effects on Diet Quality

Extreme carbohydrate restriction can profoundly affect diet quality, typically curtailing or eliminating fruits, vegetables, whole grains, and legumes and increasing consumption of animal products. Very-low-carbohydrate diets may lack vitamins, minerals, fiber, and phytochemicals found in fruits, vegetables, and whole grains (6–8). Low-carbohydrate diets are often low in thiamin, folate, vitamin A, vitamin E, vitamin B6, calcium, magnesium, iron, and potassium (9). In the absence of multivitamin supplements, individuals on low-carbohydrate diets are at risk of frank nutritional deficiencies (10). Even when consuming only nutrient-dense foods, a 4:1 ketogenic diet is reported to have multiple micronutrient shortfalls, often lacking in vitamin K, linolenic acid, and water-soluble vitamins excluding vitamin B12 (11).

Ketogenic diets are typically low in fiber needed not only for healthful intestinal function but also for microbial production of beneficial colonic short-chain fatty acids (12), which enhance nutrient absorption, stimulate the release of satiety hormones, improve immune function, and have anti-inflammatory and anti-carcinogenic effects (13, 14). Inadequate intake of these microbiota-accessible carbohydrates found in plant cell walls also increases gut permeability, as bacteria extract the carbon they need from the mucus membrane that protects the gastrointestinal tract instead of fiber (15). The relative abundance of certain health-promoting, fiber-consuming bacteria has been found to be reduced in children consuming a ketogenic diet for epilepsy (16). It has been suggested that supplementation of ketogenic diets with fiber and non-digestible carbohydrates might be advisable (16), although data to confirm that supplementation could counteract the effects of very-low-carbohydrate diets on the gut microbiota are lacking.

Intake of other protective dietary components may also be insufficient, such as phytochemicals (e.g., flavanones and anthocyanins), which are not typically included in multivitamins and for which specific intake targets have not been established. Low-carbohydrate diets are also typically high in saturated fat and cholesterol (10).

## Effects of Ketogenic Diets by Condition

### Seizure Disorders

Worldwide, the lifetime prevalence of epilepsy is 7.6 per 1,000 people (17). According to a 2018 Cochrane Review, most affected individuals can eliminate seizures with medication, but about 30% cannot. Some one-third to one-half of people with drug-resistant epilepsy can reduce seizure frequency by at least 50% with a ketogenic diet (18). The lack of glucose available to fuel neurons is a possible mechanism for action (19).

Long-term adherence is challenging, as food choices are limited and adverse effects are common (18). Micronutrient supplementation is required. Potential health risks accompany the long-term use of such a diet, as described below. Research has shown that modified versions of the ketogenic diet allowing for more carbohydrates have also been somewhat effective in seizure reduction (19). Most studies have not been long term, large scale, nor conducted with adult participants; therefore, more research is needed.

### Obesity and Weight Management

Ketogenic diets can induce weight loss (20–23). In a 2020 meta-analysis of 38 studies lasting 6–12 months and including 6,499 participants, low-carbohydrate diets, defined here as <40% of energy from carbohydrate, led to a small weight loss, compared with low-fat diets, defined as <30% of energy from fat (mean difference −1.30 kg; 95% CI, −2.02 to −0.57), with considerable variability between individuals and between studies. More than half of included studies met criteria for a general ketogenic diet, as defined in Table 1, for part or all of the low-carbohydrate intervention (24).

It has been proposed that weight loss on ketogenic diets may be due to reduced appetite (25), an effect also seen in those following balanced, very-low-energy diets (<800 kcal/day). Since ketosis occurs on both types of diets, though to a lesser degree with very-low-energy diets, it is speculated that ketosis itself may decrease hunger (26). However, findings from a recent trial by Hall et al. suggest that a low-fat vegan diet (10% energy from fat) may be more effective than a ketogenic diet in suppressing appetite (27). Energy expenditure has also been shown to increase on a ketogenic diet, at least in short-term studies (27, 28).

In controlled trials, low-carbohydrate diets appear no more effective than other diets that similarly restrict calories (29), nor are they more effective than other dietary interventions, such as low-fat vegetarian diets, at inducing weight loss (30, 31). A 2013 meta-analysis of randomized controlled trials testing very-low-carbohydrate ketogenic diets (≤50 g carbohydrate/day or ≤10% kcal from carbohydrates) against diets based on modest reductions in fat intake (<30% kcal from fat) for at least 1 year found that ketogenic diets led to marginally more weight loss than reduced-fat diets (weighted mean difference: −0.91 kg; 95% CI, −1.65 kg to −0.17 kg, *p* = 0.02). However, no statistically significant difference in amount of weight lost was seen between the 2 diets in trials following people for at least 2 years (3).

A 2017 meta-analysis of 9 trials echoed these findings. In studies <12 months long, low-carbohydrate diets (<130 g carbohydrate/day or <26% kcal from carbohydrates) were seen to lead to greater weight loss in people with type 2 diabetes relative to normal- or high-carbohydrate control diets (weighted mean difference: −1.18 kg; 95% CI, −2.32 kg to −0.04 kg; *p* = 0.04). No advantage was seen relative to control diets in studies of longer duration (weighted mean difference: −0.24 kg; 95% CI, −2.18 kg to 1.7 kg; *p* = 0.81) (32).

At least initially, ketogenic diets may slow fat loss. In a 2016 metabolic ward study by Hall et al., 17 overweight or obese men were provided a baseline diet (50% carbohydrate, 35% fat, and 15% protein, as a percent of energy) for 4 weeks, then a ketogenic diet (5% carbohydrate, 80% fat, 15% protein) for 4 weeks. For 2 weeks after switching from the baseline diet to the ketogenic diet, participants' weight loss accelerated—but fat loss slowed. The authors attributed the additional weight loss primarily to loss of body water. However, loss of body protein may have contributed; urinary nitrogen levels increased through day 11 on the ketogenic diet. In the final 2 weeks on the ketogenic diet, participants' rates of body weight and fat loss rebounded to a rate comparable to that on the baseline diet. As a result, study participants required 4 weeks on a ketogenic diet to lose the same average 0.5 kg of fat lost in the final 2 weeks on a baseline diet. It is not clear whether these effects have longer-term consequences (28).

The 2021 metabolic ward study by Hall et al. tested the effects of both an animal-based ketogenic diet (76% energy from fat, 10% carbohydrate) and a plant-based, low-fat diet (75% carbohydrate, 10% fat) on 20 weight-stable adults, mean age 29.9 years, mean BMI 27.8 kg/m^2^ (27). Participants were randomized to each diet, which they consumed *ad libitum* for 2 weeks before immediately crossing over to the other diet. *Ad libitum* energy intake was 689 kcal/day lower on the low-fat, plant-based diet as compared to the ketogenic diet (*p* < 0.0001). Reported hunger and satisfaction were similar between groups. Both diets induced weight loss: 1.77 ± 0.32 kg (*p* < 0.0001) for the ketogenic diet vs. 1.09 ± 0.32 kg (*p* = 0.003) for the low-fat diet. However, most of the weight lost on the ketogenic diet came from fat-free mass (-1.61 ± 0.27 kg; *p* < 0.0001); this was not the case with the low-fat diet (−0.16 ± 0.27 kg; *p* = 0.56). Fat mass did not significantly change during either the first or second week of the ketogenic diet, while the low-fat diet led to significant losses in body fat after both the first and second weeks. This suggests that low-fat, plant-based diets may control appetite better than ketogenic diets. These results also add to evidence suggesting that the rapid initial weight loss observed on ketogenic diets is due predominantly to loss of fat-free mass (e.g., body water, glycogen, protein, and contents of the gastrointestinal tract) (27).

### Diabetes

#### Type 1 Diabetes

Although ketogenic diets can improve glycemia in pediatric patients with type 1 diabetes, they are generally not used in this population due to the risk of malnutrition, failure to thrive, reduced bone density, hyperlipidemia, poor sleep, amenorrhea, and hypoglycemia. In addition, mood and behavior may be adversely affected (33).

In adults with type 1 diabetes, both favorable and unfavorable outcomes have been observed. A small study of 11 adults with type 1 diabetes reported that a ketogenic diet improved blood glucose control (34). However, the ketogenic diet triggered more frequent and extreme hypoglycemic episodes (6.3 episodes per week compared to 1–2 episodes per week typically reported for those following conventional or otherwise unspecified diets). The majority of participants also developed dyslipidemia. Lipid changes are of particular concern in individuals with diabetes, who are already at heightened risk of cardiovascular events (34).

A comprehensive review strongly discouraged sustained ketosis or hyperketonemia in individuals with type 1 diabetes (35). Due to metabolic irregularities associated with type 1 diabetes, ketone production is elevated, and ketone clearance is diminished. Individuals with elevated ketones are at increased risk for complications of the microvasculature, brain, kidney, and liver compared to those with normal ketone levels. In type 1 diabetes, hyperketonemia is associated with oxidative stress, inflammation, non-alcoholic fatty liver disease, and insulin resistance (35).

#### Type 2 Diabetes

##### Management

Ketogenic diets depress appetite, promote weight loss, reduce blood glucose values, and decrease HbA1c in the short term (21, 36–43). Some studies have reported improved insulin sensitivity (40); the effect appears to be dependent on loss of fat mass (44). In the abovementioned metabolic ward study in which 17 overweight or obese men were provided a baseline diet (50% carbohydrate) for 4 weeks and then a ketogenic diet (5% carbohydrate) for 4 weeks, during the ketogenic diet phase, total cholesterol, low-density lipoprotein cholesterol (LDL-C), and C-reactive protein increased significantly, while fasting insulin and triglycerides decreased. While on the ketogenic diet, insulin sensitivity was impaired when participants were challenged with a baseline diet meal (50% carbohydrates) (45).

In the 2021 metabolic ward trial by Hall et al. comparing the effects of an animal-based ketogenic diet and a plant-based, low-fat diet, the plant-based diet had a greater glycemic load and predictably resulted in higher postprandial glucose and insulin levels than the ketogenic diet. However, glucose tolerance, as determined by an oral glucose tolerance test at the end of each phase, was compromised during the ketogenic phase (average 2-h glucose was 142.6 mg/dL) compared to the plant-based phase (average 2-h blood glucose was 108.5 mg/dL). In addition, high-sensitivity C-reactive protein, a marker of inflammation, was substantially higher while on the ketogenic diet compared to the plant-based diet (2.1 vs. 1.2 mg/L; *p* = 0.003), although not significantly different from baseline (27).

Another low-carbohydrate diet trial that followed individuals for 1 year found that insulin sensitivity was improved at 6 months but returned to baseline at 1 year (22). In healthy men, a ketogenic diet (83% fat and 2% carbohydrate) reduced insulin's ability to suppress endogenous glucose production (46).

A recent meta-analysis showed that reductions in hemoglobin A1c achieved with carbohydrate-restricted diets typically wane after a few months and that such diets are not more effective than other diets (47).

In other clinical trials with ketogenic diets, diabetes medications are frequently reduced or eliminated (21, 36–43). The beneficial effects of ketogenic diets for people with type 2 diabetes are attributable primarily to weight loss, with benefits appearing to wane over time (48, 49). Few additional negative impacts on global measures of health have been reported in short-term studies on type 2 diabetes (21, 37, 40). Long-term effects have not been elucidated (49).

##### Prevention

The prospective Nurses' Health Study found no link between diets lower in carbohydrate and incident type 2 diabetes in women, although those consuming the most vegetable protein and fat had an 18% lower risk (50). The Health Professionals Follow-Up Study found that men consuming diets low in carbohydrate and high in animal protein and fat had a 37% higher risk of being diagnosed with type 2 diabetes than those who scored lowest for this diet style. Those emphasizing vegetable protein and fat on low-carbohydrate diets did not experience increased risk, and for men under 65 years of age, diabetes risk was 22% lower (51).

Dietary staples in ketogenic diets include concentrated fats, meat, poultry, fish, eggs, and cheese, all of which have been associated with increased diabetes risk (52–56). These foods can be high in saturated fat, cholesterol, chemical contaminants, pro-oxidants such as heme iron, and inflammatory compounds such as N-glycolylneuraminic acid (Neu5Gc) and endotoxins. Conversely, foods consistently associated with reduced diabetes risk, including fruits, legumes, whole grains, and several vegetables, are minimized or eliminated (52–56).

### Non-alcoholic Fatty Liver Disease

Non-alcoholic fatty liver disease (NAFLD) is a serious condition where excess fat is stored in hepatocytes, causing steatosis, which can progress to non-alcoholic steatohepatitis and increase the risk of hepatocellular carcinoma (57–60). Worldwide, the prevalence of NAFLD in adults is estimated to be 25.2%, ranging from a low of 13.5% in Africa to a high of 31.8% in the Middle East, with North America at 24.1% (61). The risk of NAFLD is significantly higher in individuals who have obesity or type 2 diabetes (43–92%) (57, 58, 62).

Hepatic triacylglycerol comes from three sources: *de novo* lipogenesis, primarily from glucose; lipolysis of stored triglyceride from adipose tissue; and diet-derived fats (58). Most (60–80%) triglyceride is from adipose tissue, 15% is from diet, and 5% is from *de novo* lipogenesis in healthy people. Triglyceride from *de novo* lipogenesis is much higher (26%) in individuals with NAFLD (63). Fat derived from *de novo* lipogenesis and adipose tissue is accelerated by insulin resistance (63).

Several clinical trials have compared low-fat and low-carbohydrate hypocaloric diets in overweight or obese adults and found similar reductions in intrahepatic fat (64–66). Ketogenic diets typically increase intake of saturated fat, cholesterol, and animal protein, all of which are associated with insulin resistance, oxidative stress, and an exacerbated flow of free fatty acids to hepatocytes (57, 62, 63, 67).

In epidemiological studies, diets high in saturated fat, *trans* fat, simple sugars, and animal protein (especially from red and processed meat) (57) and low in dietary fiber and omega-3 fatty acids (62, 68) are thought to contribute to NAFLD. In the Rotterdam Study, those consuming the most animal protein were 54% more likely to have NAFLD than those consuming the least (OR 1.54, 95% CI, 1.20–1.98) (68). Dietary components associated with reduced NAFLD risk include whole grains, nuts and seeds, monounsaturated fats, omega-3 fatty acids, vegetable protein, prebiotic fiber, probiotics, resveratrol, coffee, taurine, and choline (57). In the Tzu Chi Health Study, replacing one serving of soy with fish (or meat) was associated with a 12–13% increased risk of NAFLD. Whole grain intake had an inverse relationship with NAFLD, and those following a vegetarian diet had a 21% lower risk of NAFLD (69).

Lifestyle modifications, particularly diet change, weight loss, and exercise, are the primary modality for treating NAFLD (57, 62, 63). Lifestyle interventions that promote weight loss have been found to reduce liver fat and improve aminotransferase concentrations and insulin sensitivity (48, 57, 58, 62, 63, 68). It has been suggested that achieving ketosis may have a benefit in ameliorating fatty liver (63), but the studies supporting this are limited and typically also restrict energy intake. Long-term safety and specific clinical outcomes have not been determined.

### Cancer

#### Management

Some have suggested ketogenic diets for cancer patients (70) based on the so-called “Warburg effect,” whereby cancer cells increase glucose uptake and upregulate glycolysis even in the presence of oxygen, preferentially fermenting glucose to lactate (71). By nearly eliminating available glucose, ketogenic diets theoretically stress cancer cells.

Few clinical trials have tested this. A 2018 systematic review of ketogenic diets for the management of gliomas found no randomized clinical trials and just 6 published case series/reports. While the authors could not evaluate the effectiveness of ketogenic diets for cancer survival, they noted that minimal adverse events were reported, suggesting ketogenic diets may be safe in this population (72).

A 2020 systematic review analyzed 13 studies of ketogenic diets as a complementary therapy for standard treatments in a variety of cancers. Studies analyzed were small (*n* = 2–44); 9 were prospective and 6 were controlled, but just 2 were randomized, and ketogenic diet prescriptions differed between studies. Diet-related adverse events were uncommon and mostly minor, and the diet had a beneficial effect on body composition. Findings were mixed for both overall survival and progression-free survival; beneficial effects were seen in four studies (73). A possible explanation for the lack of a consistent survival benefit is demonstrated in *in vitro* research suggesting that ketone utilization by cancer cells increases expression of genes associated with high metastatic potential (74). Given potential benefits for body composition, large, well-designed, randomized clinical trials are needed to determine the safety and effectiveness of ketogenic diets in cancer treatment (72, 73).

#### Prevention

Long-term data on cancer outcomes with ketogenic diets are lacking. However, food components typical of a ketogenic diet, such as red and processed meats, are linked to increased cancer risk (75–77). Whole grains, fruits, and vegetables are linked to a lower risk of both cancer and all-cause mortality (78, 79), yet, with the exception of non-starchy vegetables, these foods are commonly avoided on ketogenic diets. For example, in one study of a ketogenic diet for type 2 diabetes, researchers encouraged unlimited meat, poultry, seafood, and eggs, while cutting intake of whole grains, fruits, and starchy vegetables and limiting intake of salad vegetables and non-starchy vegetables (21).

### Alzheimer's Disease

#### Management

By 2050, it is projected that 13.8 million people in the U.S. will have Alzheimer's disease (AD) (80). Given the brain's inability to efficiently utilize glucose in AD, some have proposed ketones as an alternate fuel source for these individuals (81). As reviewed by Włodarek in 2019, small trials have found that increasing blood ketones by supplementing with medium-chain triglycerides does improve some measures of cognitive function in AD, although not necessarily in those with the APOEε4 genotype (82).

No long-term data on ketogenic diets for AD are available, although small, short-term trials have been conducted. A 3-month, weight-maintaining ketogenic diet intervention improved cognition in subjects with mild-to-moderate AD (*n* = 15), but improvements were lost after a 1-month washout period (83). A 6-week trial of a ketogenic diet in subjects with mild cognitive impairment led to improved memory relative to a control diet (50% of energy from carbohydrates); follow-up data were not available. However, the ketogenic diet was substantially lower in calories, which may have independently reduced insulin resistance (84). In a 2020 review of short-term ketogenic diet and ketone supplement studies in older adults, including those with no dysfunction, mild cognitive impairment, and AD, 6 of 9 controlled trials with clinical endpoints found significant cognitive improvements in the intervention groups, while other trials did not. Whether cognitive gains would be maintained upon discontinuation of the diet/supplement remains unknown due to lack of long-term follow-up (85).

#### Prevention

Saturated fat intake, which typically increases on a ketogenic diet, is strongly associated with AD risk. In the Chicago Health and Aging Project, high saturated fat intake was linked to a 2- to 3-fold increased risk of incident AD (86). A 2016 review of international data found that consuming meats, eggs, high-fat dairy such as butter and cheese, and sweets was linked to an increased risk of AD (87). Aside from sweets, consumption of these foods generally increases on a ketogenic diet.

Polyphenol-rich plant foods such as fruits and vegetables are associated with lower AD risk (88) and diets focusing on whole plant foods and limiting animal foods and processed foods, such as the MIND diet, are proven to reduce AD risk (89). Thus, by providing ketones that can be metabolized by neurons in AD, a ketogenic diet could improve symptoms in the short term, but the diet's nutritional profile could increase risk over the long-term in healthy individuals.

### Cardiovascular Disease

The effect of low-carbohydrate diets on plasma lipid concentrations is a major concern. It has long been established that weight loss by any means causes a reduction in total cholesterol of about 2 mg/dL per kilogram lost (90). However, low-carbohydrate diets are often an exception to that rule. In a 2002 6-month study of a very-low-carbohydrate “Atkins” diet by Westman et al., 12 (29%) of the 41 participants had LDL-C elevations. The average increase was 18 mg/dL (91). In a similar 6-month study by Yancy, 30% of participants had LDL-C increases > 10% (92).

In a trial published in 2003 by Foster et al., LDL-C rose 6.2% in a group of low-carbohydrate dieters at 3 months (22). For comparison, LDL-C dropped by 11.1% during this same time period in participants following a conventional low-calorie diet. In a 2004 1-year study, those on a low-carbohydrate diet increased their mean LDL-C from 112 to 120 mg/dL (93). In 2018, Hallberg (94) reported a mean 10% rise in LDL-C in individuals following low-carbohydrate diets, an elevation that persisted during 2 years of follow-up (95). A recent meta-analysis of 5 studies showed that, in individuals with type 2 diabetes, ketogenic diets led to, on average, no substantial change in LDL-C (96).

It is important to note that changes reported in group means do not reflect the change for any given individual. In the 2002 study cited above, while the mean LDL-C increase was 18 mg/dL, one participant's LDL-C concentration increased from 123 to 225 mg/dL (91). In the Yancy study, one participant's LDL-C increased from to 219 mg/dL. Another experienced an LDL-C rise from 184 to 283 mg/dL, and a third developed chest pain and was subsequently diagnosed with coronary heart disease (92). In the Foster study, the standard deviation for the change in LDL-C was 20.4%, indicating that while LDL-C decreased for some, for many participants, LDL-C rose dramatically (22).

Negative effects on blood lipids have also been seen in healthy individuals. A 2018 pilot study of young, fit adults (average age 31) found that 12 weeks on a ketogenic diet led to a weight loss of 3.0 kg in the ketogenic group, with no significant weight change in the control group. However, despite significant weight loss, LDL-C increased by 35% in the ketogenic group (*p* = 0.048), from 114 mg/dL at baseline to 154 mg/dL at 12 weeks (97).

Some have suggested that LDL-C or LDL particle concentration elevations are of no concern if the increase is mainly in larger LDL particles. There are two problems with this rationale: First is the problem of heterogeneity noted above (i.e., individuals may have significant worsening of their lipid profiles that are not reflected by mean figures). Second, LDL is potentially atherogenic regardless of particle size (98, 99). Data supporting this concern come from the Women's Health Study, a randomized, placebo-controlled trial of low-dose aspirin and vitamin E. As part of the study, LDL particle size was assessed. The hazard ratio for incident cardiovascular disease associated with large LDL particles was 1.44 (indicating a 44% increased risk). For small LDL, it was 1.63 (a 63% increased risk). Both were highly statistically significant. In other words, large LDL particles were strongly atherogenic, albeit less so than small LDL (100).

It has also been proposed that the risk elevation associated with increased LDL-C concentrations may be neutralized to the extent that high-density lipoprotein cholesterol (HDL-C) also rises. However, both Mendelian randomization trials and studies using HDL-elevating agents have not shown benefit regarding cardiovascular risk. In the former category are studies that have examined individuals with naturally occurring genetic variants associated with elevated plasma HDL-C concentrations. These genetic traits are not associated with reduced risk of myocardial infarction unless they also reduce LDL-C (101).

Treatment-induced HDL-C elevations were examined in a meta-analysis of 108 studies including 299,310 participants, which found no associated reduction in the risk of coronary heart disease events, coronary disease mortality, or total mortality (102). The LDL-C/HDL-C ratio was not a better predictor of cardiovascular outcomes than LDL-C alone, and the authors recommended using LDL-C, rather than HDL-C or a ratio of the two, as the therapeutic target.

### Kidney Health

The evidence of the renal-specific effects of ketogenic diets is limited but worth noting, especially in the context of the unclear long-term benefits of such diets for diabetes and obesity (103). For those without chronic kidney disease (CKD), one of the biggest potential risks of the ketogenic diet is the development of kidney stones, a finding that has been frequently noted in the pediatric epilepsy literature (104, 105). The ketogenic diet's emphasis on high-fat, animal-based foods while excluding many fruits and vegetables promotes a urinary milieu for kidney stones. Dietary animal protein consumption is a well-established promoter of kidney stones (106). The acidosis caused by the ketogenic diet may also encourage stone formation by lowering urinary citrate and pH levels while increasing urinary calcium levels.

Another potential risk of animal-based ketogenic diets for those without CKD is the development of CKD through the consumption of animal fat and protein. In observational studies of populations eating Western diets, high animal fat consumption, as is common with ketogenic diets, has been associated with increased risk of developing albuminuria (107). In a prospective study of nearly 12,000 people over 23 years, high animal protein consumption was associated with a 23% increased hazard ratio of incident CKD (108). Other observational studies of animal protein have shown similar findings (109, 110).

For those with CKD, the high protein content in some ketogenic diets is of concern. While “classic” ketogenic diets are not necessarily high in protein, those used for weight loss often meet the definition of a high-protein diet (>1.5 g/kg/d) by encouraging dieters to consume 1.2–2.0 g/kg/d. Compared to control diets with higher protein content, low protein consumption has been associated with a reduction in the rate of kidney function decline in a meta-analysis of 14 randomized controlled trials (111). High protein consumption facilitates hyperfiltration, a phenomenon of increased blood flow to the glomerulus, which is thought to lead to long-term damage in those with CKD (112). Finally, the acid load from the ketogenic diet may worsen metabolic acidosis and kidney disease in those with CKD (113). The ketogenic diet's acid load comes from the foods consumed (especially those from animal-based sources), ketoacids associated with ketone production, and from the lack of natural alkali found in fruits and vegetables that are often avoided in the ketogenic diet. As such, the ketogenic diet requires further research regarding its long-term renal safety in those with and without CKD.

### Pre-pregnancy and Pregnancy

Approximately 40% of pregnancies in the United States are unplanned (114). Low-carbohydrate diets followed prior to conception or during the periconceptual period are associated with an increased risk of birth defects and gestational diabetes, respectively.

The National Birth Defects Prevention Study found that women who reported consuming low-carbohydrate diets in the year prior to conception (daily carbohydrate intake ≤5th percentile of control mothers, or ~95 g carbohydrate/day) were 30% more likely to have an infant with a neural tube defect (95% CI, 1.02–1.67), specifically anencephaly (OR 1.35; 95% CI, 0.90–2.02) and spina bifida (OR 1.28; 95% CI, 0.95–1.72) (115). For unplanned pregnancies in particular, effect estimates for carbohydrate-restricted diets showed an 89% increased risk of neural tube defects (95% CI, 1.28–2.79) (115).

Use of folate supplements may not mitigate the risk seen with low-carbohydrate diets. In the above study, there was no effect measure modification by folic acid supplement use (115). A 2019 study conducted using data that predated the era of folate-fortified grain products also found an increase in neural tube defects in the offspring of women consuming low-carbohydrate diets in the periconceptual period (OR 2.0; 95% CI, 1.2–3.4), suggesting other factors were contributing (116).

A prospective cohort study evaluating gestational diabetes risk scored women's diets for adherence to a low-carbohydrate diet pattern and dietary fat source. After adjusting for multiple variables including BMI, women consuming the least carbohydrate had a 27% higher risk of gestational diabetes compared to those consuming the most (RR 1.27; 95% CI, 1.06–1.51, *p* = 0.03). A stronger association was seen for women following a low-carbohydrate diet pattern high in animal products; they had a 36% higher risk of gestational diabetes (RR 1.36; 95% CI, 1.13–1.64, *p* = 0.003). A vegetable-based low-carbohydrate dietary pattern was not associated with increased risk (117).

## Adverse Effects of Ketogentic Diets

The most restrictive ketogenic diets used for epilepsy can cause fatigue, headache, nausea, constipation, hypoglycemia, and acidosis, especially within the first few days to weeks of following the diet (2). Dehydration, hepatitis, pancreatitis, hypertriglyceridemia, hyperuricemia, hypercholesterolemia, hypomagnesemia, and hyponatremia can also occur (82, 118).

A study of 300 users of online forums found that self-administered ketogenic diets may be accompanied by a temporary cluster of symptoms frequently termed “keto flu,” which includes headache, fatigue, nausea, dizziness, “brain fog,” gastrointestinal discomfort, decreased energy, feeling faint, and heartbeat alterations (119). In endurance athletes, 3.5 weeks on a ketogenic diet led to unfavorable effects on markers of bone modeling and remodeling (120).

Longer-term effects can include decreased bone mineral density, nephrolithiasis, cardiomyopathy, anemia, and neuropathy of the optic nerve (82, 121). Ketogenic diets have low long-term tolerability, and are not sustainable for many individuals (48, 49). Diets low in carbohydrate have also been associated with an increased risk of all-cause mortality (122), although recent data suggest that lower-carbohydrate diets can be linked to either higher or lower mortality risk, depending on the quality of the carbohydrate they contain and whether they rely more on animal protein and saturated fat or plant protein and unsaturated fat, respectively (123).

## Conclusion

Ketogenic diets reduce seizure frequency in some individuals with drug-resistant epilepsy. These diets can also reduce body weight, although not more effectively than other dietary approaches over the long term or when matched for energy intake. Ketogenic diets can also lower blood glucose, although their efficacy typically wanes within the first few months.

Very-low-carbohydrate diets are associated with marked risks. LDL-C can rise, sometimes dramatically. Pregnant women on such diets are more likely to have a child with a neural tube defect, even when supplementing folic acid. And these diets may increase chronic disease risk: Foods and dietary components that typically increase on ketogenic diets (eg, red meat, processed meat, saturated fat) are linked to an increased risk of CKD, cardiovascular disease, cancer, diabetes, and Alzheimer's disease, whereas intake of protective foods (eg, vegetables, fruits, legumes, whole grains) typically decreases. Current evidence suggests that for most individuals, the risks of such diets outweigh the benefits.

## Author Contributions

LC and NDB contributed to the organization of the manuscript, reviewed, and approved the submitted version. LC composed the outline and drafted the manuscript. LC, BD, SJ, MJ, JP, MN, and NDB wrote sections of the manuscript. All authors had full access to data and revised and approved the manuscript for publication.

## Conflict of Interest

LC is an employee of the Physicians Committee for Responsible Medicine in Washington, DC, a non-profit organization providing educational, research, and medical services related to nutrition. LC also declares that a trust for her benefit previously held stock in 3M, Abbot Labs, AbbVie, Johnson and Johnson, Mondelez, Nestle, and Walgreens; she is the author of a food and nutrition blog, Veggie Quest; and she is former publications editor and current chair for the Women's Health Dietetic Practice Group within the Academy of Nutrition and Dietetics. MJ and JP received compensation from the Physicians Committee for Responsible Medicine while working on this manuscript. MN is an employee of the Physicians Committee for Responsible Medicine. NDB is an Adjunct Professor of Medicine at the George Washington University School of Medicine. He serves without compensation as president of the Physicians Committee for Responsible Medicine and Barnard Medical Center in Washington, DC, non-profit organizations providing educational, research, and medical services related to nutrition. He writes books and articles and gives lectures related to nutrition and health and has received royalties and honoraria from these sources. The remaining authors declare that the research was conducted in the absence of any commercial or financial relationships that could be construed as a potential conflict of interest.
